# The Expressivist Objection to Nonconsensual Neurocorrectives

**DOI:** 10.1007/s11572-021-09566-9

**Published:** 2021-04-09

**Authors:** Gabriel De Marco, Thomas Douglas

**Affiliations:** 1 The Oxford Uehiro Centre for Practical Ethics, University of Oxford, Suite 8, Littlegate House 16/17 St Ebbe’s Street, Oxford OX1 1PT, UK; 2 Jesus College, Turl Street, Oxford OX1 3DW, UK

**Keywords:** Rehabilitation, Neurolaw, Neurocorrectives, Respect, Bodily Integrity, Mental Integrity

## Abstract

Neurointerventions—interventions that physically or chemically modulate brain states—are sometimes imposed on criminal offenders for the purposes of diminishing the risk that they will recidivate, or, more generally, of facilitating their rehabilitation. One objection to the nonconsensual implementation of such interventions holds that this expresses a disrespectful message, and is thus impermissible. In this paper, we respond to this objection, focusing on the most developed version of it— that presented by Elizabeth Shaw. We consider a variety of messages that might be expressed by nonconsensual neurointerventions. Depending on the message, we argue either that such interventions do not invariably express this message, that expressing this message is not invariably disrespectful, or that the appeal to disrespect is redundant.

## 1 Introduction

Neurointerventions—interventions that physically or chemically modulate brain states—are sometimes imposed on criminal offenders for the purposes of diminishing the risk that they will recidivate, or, more generally, of facilitating their reha-bilitation.^1^ For example, in several European countries and US states, sex offenders are sometimes required to take hormonal agents that suppress their sex drive, either as part of their initial sentence, or as a condition of early release. Similarly, offenders addicted to opioid drugs are sometimes required to receive methadone maintenance therapy intended to suppress their desire for their substance of addiction. Some speculate that, in the future, violent offenders might be required to receive aggression- or impulsivity-attenuating drugs, and that psychopathic offenders might be required to undergo pharmaceutical or neurostimulatory interventions intended to enhance their capacity for empathy or sympathy.^2^ These are examples of what we will call *neurocorrectives*: neurointerventions administered to criminal offenders for the purposes of preventing their recidivism or promoting their rehabilitation.

In a series of recent articles, Elizabeth Shaw has developed a rich and nuanced critique of neurocorrectives, focusing especially—though not exclusively—on cases where they are used without the valid consent of the offender. This would be the case where, for example, the neurocorrective is imposed as a mandatory element of a criminal sentence.^3^ We will call these *Nonconsensual Neurointerventions*, or NNs for short. There are several strands to Shaw’s critique. One of these holds that imposing NNs is, at least in many cases, impermissible by virtue of infringing the offenders’ rights to bodily and/or mental integrity (Shaw 2018a, 2018b, 2019a). Call this the Rights-Based Objection. Another—and the one we will focus on here— holds, first, that NNs invariably express disrespect:

mandatory neurointerventions communicate disrespect, because of the social meaning that attaches to violations of the rights to mental and bodily integrity (Shaw 2018a, p. 328).

And second, that NNs are invariably wrong by virtue of expressing this disrespect:

[the] disrespect that is expressed … renders mandatory neurointerventions impermissible (Shaw 2018a, p. 336).

Call this the Expressivist Objection.^4^

The Expressivist Objection is not idiosyncratic. Similar objections have long pedigree in penal theory,^5^ and Shaw is not the only author to have advanced expressivist or disrespect-based objections against NNs (Bennett 2018; Bublitz 2018; Bublitz and Merkel 2014; Kirchmair 2019).^6^ However, we focus on Shaw’s objection as she has developed it more fully than others.

As Shaw characterizes it, the Expressivist Objection is capable of explaining why NNs are impermissible even when they do not impermissibly infringe rights to bodily or mental integrity.^7^ The Objection

depends on this reason: expressed disrespect. I do not claim that the *fundamental nature* of the rights to bodily/mental integrity on its own (without the element of expressed disrespect) can explain why all mandatory neurointerventions are wrong (Shaw 2018a, p. 323, emphasis in original).^8^

Here, we take no stance on the Rights-Based Objection. We instead argue against the Expressivist Objection—the objection that NNs are invariably wrong because they are invariably disrespectful. We will consider a variety of messages potentially expressed by NNs, divided into two types: status messages, and capacity messages. Status messages express a message about the subject’s moral status, whereas capacity messages merely express a message about the subject’s capacities. Depending on the message, we will argue either that NNs do not invariably express this message, that expressing this message is not invariably disrespectful, or that the appeal to disrespect is redundant.

Before presenting our arguments, we offer some clarificatory comments.

The first concerns the meaning of ‘disrespect’. Drawing on Jean Hampton’s work, Shaw understands disrespectful treatment as treatment “whose meaning appropriately understood by members of the cultural community in which the behaviour occurs, represents her value as less than the value she should be accorded” (Hampton 1991, p. 1670, cited in Shaw 2018a, p. 322; 2019b, p. 7).^9^ In the interests of charity, we will understand ‘disrespectful treatment’ somewhat more broadly, as treatment that expresses a negative misrepresentation concerning the offender, without requiring that the misrepresentation concern the *value* of the victim.^10^ Following Shaw, we will sometimes speak of “expressed disrespect” and “disrespectful messages.” Such claims are intended to follow this notion of disrespect; i.e., a disrespectful message is one that expresses a negative misrepresentation concerning the offender, and if there is expressed disrespect, this is because a disrespectful message is expressed.

Given that the content of the message expressed depends on how the action will be appropriately understood by members of the community, what message is expressed will be partly an empirical matter. However, we do not know of any empirical research that provides clear and strong evidence on how such actions would be interpreted, and in any case, such evidence would not, by itself, tell us whether the understanding of the action is *appropriate*. Thus, our approach will be to consider a variety of messages that might be expressed by NNs and that are somewhat conducive to Shaw’s view. As we explain in Sect. 3, our core argument could also be extended to many other potential messages that NNs might express.

One might worry that the arguments presented here only work against a version of the Expressivist Objection that relies on this particular notion of disrespect. Variants of the Objection relying on a different understanding of disrespect may be able to evade our arguments. For instance, on a different understanding of disrespect, an action can express disrespect in virtue of being motivated by a certain sort of attitude, rather than in virtue of expressing a certain sort of message. If the attitude is of the right sort, then the action is disrespectful. One of us has previously argued against this form of Expressivist Objection to NNs (Douglas 2014, 2019), so we will not consider it further here, though we do briefly comment on the prospects for other variants of the Expressivist Objection in the conclusion.

Finally, a comment on why expressing disrespect might be wrong. One reason that it might be wrong is that it causes harm to the person who is disrespected—or other relevantly similar people. Shaw draws attention to these harmful effects (Shaw 2011, pp. 198–201; 2014, pp. 12–13; 2019a, p. 105). However, she does not limit the scope of her objection to cases in which these effects will in fact occur, or can reasonably be expected to occur.^11^ She appears to believe that expressing a negative misrepresentation about someone, through administering an NN, is wrong even in the absence of (an expectation of) harm. Her thought, we take it, is that there are moral reasons to refrain from expressing the sort of negative misrepresentation sent by NNs, and these reasons are present in virtue of some *intrinsic feature* of the expression. We will likewise take it that the success of the Expressivist Objection does not depend on whether expressing disrespect causes harm.

## 2 Capacity Messages

On a capacity message interpretation of the Expressivist Objection, NNs express the message that the offender lacks some capacity or that they have it to a low degree. This may be the thought behind the claims that using NNs would send out the message that:

all offenders who are offered the intervention stand in need of it, whether or not they ultimately disagree with it (Shaw 2014, p. 13) offenders are radically defective with regard to one of the most fundamental aspects of their agency (Shaw 2014, p. 13) [i]nvading the profoundly intimate sphere of the offender’s mind and body, depriving a person of control over *herself*, seems to convey the objectionable message that this person is fundamentally inferior and needs to be remoulded. (Shaw 2018a, p. 324)

The third statement might be read as referring to either a capacity message or a status message (or both). For the purposes of this section, we will interpret it as referring to the former: the message expressed is that the offender is inferior with regard to one or more of his *capacities*. These three claims leave it open how we are to understand the particular deficit, or what fundamental aspect of agency the offenders are purportedly missing, according to the message. One might think, for instance, that NNs express the message that the offender is not rational (the ‘rationality message’), or that the offender does not have control over his conduct (the ‘control message’). Weaker versions of these messages would merely express the message that the subject’s rationality and/or control are *limited*. For brevity, we focus on the rationality message; however, points similar to those made here apply to the control message as well.^12^

The rationality message can take various forms. It may, for instance, claim that the agent is defective in her ability to recognize reasons (reasons-receptivity), or in her ability to choose on the basis of those recognized reasons (reasons-reactivity).^13^ The rationality message that is most plausibly sent by the administration of an NN is the message that the offender’s rationality is significantly faulty with regard to at least one rational ability, without a commitment to which ability this is.

It is implausible that current, or soon to be available, NNs would express the message that the offender lacks these abilities with regard to all reasons; that is, that the offender is, at least, significantly less receptive and/or reactive than typical agents to reasons in general. Consider the fact that neurocorrectives, including nonconsensual neurocorrectives, are typically used in response to particular, rather narrowly defined types of offenses, and target particular mental states. For example, chemical castration is often targeted at those who suffer from paraphilias and have offended against children, and aims specifically at attenuating the offender’s sexual urges. This suggests that any rationality message sent by chemical castration will be quite narrow in scope. For instance, the message may be that the agent does not have the capacity to recognize or react to a specific set of reasons—say, some reasons relating to sexual conduct—at least in certain contexts. We use the term ‘local rationality messages’ or ‘LRMs’ to refer to rationality messages that—like those just mentioned—apply only to capacities concerning a specific, narrow class of reasons and/or contexts.

Supposing that NNs do, or would, express one of the LRMs mentioned above, it is not clear that there is something intrinsic to such an expression that gives us reason not to use NNs. For one thing, the message might be true, and thus not disrespectful; disrespect requires a negative *mis* representation.

Of course, one might worry that the state would use NNs without being justified in believing that the offender does have one of these flaws. However, though this is possible, it is not inevitable. Suppose that NNs were used only on individuals who have multiple convictions for the same serious crime, and following thorough and repeated psychological testing revealing cognitive deficits. In these cases, at least, it might seem that the state could have good justification for the belief that the offender suffers from a rational flaw.

There are other rational capacities that might be implicated in an LRM. One is the offender’s capacity to make the right decision concerning whether he or she should attempt to correct the rational flaw that led to the offense. Another is the capacity to actually make this correction. Perhaps NNs express the LRM that the offender lacks one, or both, of these capacities.^14^ This LRM might be what Shaw has in mind when she states that:

A policy employing direct interventions to re-shape offenders’ values would be based on the assumption that these offenders’ existing capacities for moral agency are so fundamentally inferior to the capacities of the rest of the moral community that these offenders *will not respond appropriately to the most compelling reasons for changing their behaviour*. (Shaw 2011, p. 201, emphasis ours)

This message would imply a deeper flaw in the agent’s rationality, in comparison to the LRMs discussed above. Those LRMs implied something about the offender’s capacity to recognize and/or react to a specific set of reasons in certain contexts. By contrast, the present LRM concerns the offender’s higher order rational abilities, in a broader set of contexts. It might imply that the agent is either incapable of recognizing that they did something wrong, in retrospect and outside of that context, and after having this pointed out to them by the state and a jury of their peers, or that they are incapable of transforming this recognition into behavior that results in a character change.

Again, however, even if NNs express this message, the message would not be a misrepresentation if it were true, and thus, the NN would not be disrespectful. Moreover, though it is again possible that the state might send such a message without having justification for the view that the offender has the rational flaw, it need not lack such justification. Consider a case in which an NN is imposed as a last resort, only after several other attempts at rehabilitation have failed. In this case, the state would perhaps be justified in believing that the content of the message is true— i.e., that the offender indeed lacks the ability to make the right decision concerning whether he or she should attempt to correct the rational flaw that led to the offense.

It is difficult to identify an LRM such that it is plausible both that (i) NNs always send this LRM, and (ii) sending the LRM is always disrespectful. Consequently, it is doubtful that the Expressivist Objection will be successful if it relies on a capacity message, since it is doubtful that NNs will always express a disrespectful capacity message. We now turn to consider a different type of negative misrepresentation that might be expressed by NNs: what we will call a negative status message.

## 3 Status Messages

Status messages concern the moral status of the subject; for example, the rights or non-instrumental value of the subject. Thus, to express the message that the subject has less value than a typical person, or that the subject has fewer rights (or weaker rights) than the typical person would be to express a status message. In what follows, we assume, for simplicity, that status messages are about rights.^15^

One status message that might be expressed by NNs concerns the subject’s right to be listened to. Shaw suggests that the use of NNs would portray offenders “as a group to whom we need not listen (at least not until we have modified them to ensure they will tell us what we want to hear)” (Shaw 2014, p. 13).^16^ On one interpretation of this right, it includes the right both to offer one’s own account and appraisal of one’s criminal conduct, and to present one’s views about how one should be treated by the criminal justice system. If we think of NNs as ‘silencing’ the offender, we might think they express the message that the offender does not have this right.

An initial response to this would point out the fact that offenders are afforded opportunities to state, and have heard, their case through commonplace fair trial provisions; a state policy of using NNs *following a fair trial* would seem to acknowledge that the offender has a right to be listened to.

However, one might think that the right to be listened to is not exhausted by the opportunities provided by the state in the initial trial stage. Instead, the offender has a right to *continue* being listened to, even after a verdict has been reached. Further, one might think that the nature of the activity should be a rational dialogue. Engaging the offender in dialogue recognizes “that the offender (as he is, without neurological modification) may have useful insights, as other agents do” (Shaw 2011, p. 197).

Even on this understanding of the right, though, it is not plausible that NNs express the message that offenders lack it. Not only are provisions for fair trials commonplace, provisions for appeals are as well; allowing the offender further opportunities to state their case after the initial trial. Further, although it is unclear what form this rational dialogue would take, it is also unclear why the state could not provide the offender with the time and resources to exercise this opportunity to engage in rational dialogue before the use of NNs. Moreover, the offender would seem to be able to exercise this opportunity, at least to some extent, even *after* an NN has been administered. Suppose, for instance, that the state employs an aggression reducing medication on an individual charged with aggravated assault. It is not clear what, or how much, information the subject would no longer be able to communicate after this treatment.^17^

Perhaps, however, the right to be listened to should be understood as giving one more than merely the opportunity to state one’s case, or to engage in dialogue. The right to be listened to might also imply that the state’s treatment of the offender should be *responsive* to this dialogue. This right could also be interpreted in terms of control, such that the offender has a right to some control over how the state treats him or her. Given that NNs are, by definition, nonconsensual, their use would not seem to afford such control to the offender. Consequently, using NNs could express the message that the offender does not have this right.

However, we think that the state’s administration of *some* NNs is consistent with the maintenance of this rational dialogue, as well as some receptivity to the patient’s expressed wishes. Consider two different policies.

On one policy, after the state has made repeated attempts to change the mind of the offender through psychological rehabilitation programs and the offender has proved resistant to these attempts, the state could decide to implement *some* form of neurocorrective. This could be done while including the offender in discussions concerning what type of neurocorrective to implement, for example, by allowing the offender to express views regarding, and in fact influence, *which* drug should be used, *how* it should be administered, and *how often* it should be administered. On this policy, a neurocorrective is implemented, and whether a neurocorrective is implemented is not up to the offender. Yet there is still some responsiveness to the offender’s wishes.

On the second policy, the offender is consulted about his or her wishes concerning the use of neurocorrectives (on this individual). Once the offender has given his or her reasons for or against being the subject of some neurocorrective, and the state has knowledge of the offender’s wishes, the relevant individuals representing the state can come to a decision about whether to implement a neurocorrective, while taking the offender’s reasons into account. On such a policy, the offender’s objections to neurocorrectives may be sufficient to counteract the case in favor of using neurocorrectives, yet they need not be. In cases where, after taking these into account, there is not sufficient reason to refrain from administering an NN, the neurocorrective is administered.

Neither of these policies is *maximally* responsive to the offender’s wishes; neither guarantees that the offender will be treated as he wishes. Yet such policies are still *partly* responsive to the offender’s wishes, given that the treatments will (or can) differ, in at least some features, on the basis of differences between offenders’ wishes or objections. Further, both of these policies are consistent with maintaining a rational dialogue with the offender. Thus, the use of NNs need not send the message that the agent does not have a right to be listened to, on the current interpretation of the right, nor the message that the offender does not have a right to some control over how the state treats him or her.

Of course, a proponent of the Expressivist Objection might respond that implementing NNs in a way that is *somewhat but not fully* responsive to the offender’s wishes still sends an objectionable message—for example, the message that the offender does not have the right to *full* control over how he is treated, or at least that his right to full control can, in the circumstances, justifiably be infringed. However, it is not clear that administering NNs could be wrong by virtue of expressing these messages. After all, similar messages are plausibly expressed by all somewhat coercive interventions in criminal justice including, for example, probation regimens, fines, psychological rehabilitation programs, and all forms of incarceration. Thus, offering this response seems to commit the proponent of the Expressivist Objection to a radically revisionist stance on criminal justice in general; we take it that few will want to take this line.^18^

One might think that the problematic message expressed by NNs concerns not the ‘procedural’ right to be listened to, but other, substantive rights: “[n]eurointerventions interfere with rights to mental and bodily integrity” (Shaw 2018a, p. 321). As we use the terms, bodily and mental integrity refer to freedom from bodily and mental interference, respectively. One might think that NNs infringe the right to bodily integrity (RBI) in virtue of the means of administration, which typically involves an injection. One might also worry about the effects an NN has on bodily functions; say, the production of certain hormones or neurotransmitters. Likewise, one might think that NNs infringe the right to mental integrity (RMI) given that, if successful, they change the psychological dispositions of the subject. In non-offend-ers, we would not normally contemplate infringing the RBI or RMI in this fashion.

Thus, one might worry that the imposition of NNs on offenders expresses the message that, in these individuals, the RBI and RMI have been lost. Or, more plausibly, they might express the message that these rights have been weakened to the point that it would, at least setting aside expressivist considerations, be permissible to infringe them.^19^ Perhaps expressing this message is disrespectful.

One thing to note is that this message could not be about permissibility, all things considered. If it were, we would face issues with circularity. Why is the message disrespectful? Because it states that the action is permissible, all things considered, when, in fact, it is not. Why is the action not permissible? Because it expresses a disrespectful message. Consequently, we construe the message as involving a claim about the permissibility of an action, *setting expressivist considerations aside*. For brevity, we will use the term “permissible*” as shorthand for this notion of permissibility. That is, when we say that an action is permissible*, we mean to say that, taking all moral considerations into account *except* for those concerning expressed disrespect, the action is permissible. We will use “impermissible*” similarly.

To an expressivist argument adverting to the message that the RBI and RMI have been weakened to the point that it is permissible* to infringe them, we present a dilemma. Either the message expressed is true, or it is not; on both options, the argument does not seem to do any dialectical work.

Suppose the message expressed is that the RBI has been weakened to the point that the RBI is permissibly* infringed. If this message is true—that is, if it is true that the RBI is weakened to the point that it is permissibly* infringed in this case— then the message does not involve a negative misrepresentation of the subject’s status, and the action is not disrespectful, at least not in virtue of expressing this particular message. If the action is not disrespectful, then the expressivist argument loses a crucial premise.

Suppose instead that the message is false; that is, suppose that the RBI is impermissibly* infringed, either because it has not been weakened, or not sufficiently weakened. By expressing the message that the right is permissibly* infringed, when it is in fact not, the message would be a negative misrepresentation of the subject’s status, and the action would thereby be disrespectful. The problem for the expressivist argument on this horn is that in order for the argument to be convincing, the proponent will need to argue that the message is, in fact, a negative *mis* representation. Thus, the proponent of the expressivist argument will need to argue that the infringement of the RBI, in this case, is impermissible even leaving aside expressivist considerations.

Yet, once we have an argument for this claim, the expressivist argument becomes redundant: the conclusion of the argument, that NNs are impermissible, is something that the proponent needs to argue for in order to establish the premise claiming that NNs are disrespectful.^20^

This dilemma generalizes. Not only does it apply to the RBI, but also the RMI. In fact, this dilemma seems to apply to status objections in general.^21^ If the message expressed is that the right is permissibly* infringed, and the right is, in fact, permissibly* infringed, then we don’t have a negative misrepresentation of status. If, on the other hand, the right is impermissibly* infringed, yet the message expressed claims that it is permissibly* infringed, we have a misrepresentation. But in order to argue for this, the expressivist needs to argue that the right is impermissibly* infringed, making the expressivist argument redundant.

In the next section, we consider four possible attempts to escape this dilemma, in each case briefly outlining the attempt before offering our response.

## 4 Escaping the Dilemma

### 4.1 Attempt 1

The first attempt to escape the dilemma holds that the redundancy mentioned above, in describing the second horn of the dilemma, only applies to some NNs. To explain why, it will be helpful to bring to the fore a feature of Shaw’s view that we have thus far neglected: Shaw believes that actions can acquire a meaning simply by being actions that are of the same type and part of the same social practice as other actions with that meaning (Shaw 2018a, p. 323).^22^ Thus, meanings can ‘expand’ from a narrower set of actions—which we might call the ‘meaning-setting actions’—to a broader set. Call this thesis *the Expansion Thesis*.

It might seem that the redundancy mentioned above only applies to those NNs that set the meaning of the message expressed. These would be the NNs that express the message that some right (such as the RBI or RMI) is permissibly* infringed, when in fact it is impermissibly* infringed. We suggested that the expressivist argument would be redundant in such cases since its proponent would have to argue that the right is in fact impermissibly* infringed, and that would already constitute a decisive objection to the NN. However, note that once we accept the Expansion Thesis, we open the door to the possibility that the message—that some right has been weakened to the point that it is permissibly* infringed—could expand out to other NNs; NNs that do not impermissibly* infringe the right, and in relation to which the Expressivist Objection is thus not redundant. Call these types of NNs permissible* NNs.

### 4.2 Response

We focus on the RMI as an example for our response, yet what we say here applies to other rights as well. We grant, for the sake of argument, that at least some past NNs were impermissible* by virtue of infringing the RMI. We grant also that these NNs expressed disrespect since they negatively misrepresented the strength of the offenders’ RMI: they expressed the (false) message that the RMI had been weakened to the point that it could be permissibly* infringed. Shaw may claim that the disrespect expressed by these NNs expands to other NNs: those which in fact do not infringe, or only permissibly* infringe, the RMI. However, even if we grant that the message sent by past impermissible* NNs expands to permissible* NNs, it does not follow that the *disrespect* expands. This is because, even if permissible* NNs express a message with the same content as impermissible* NNs, in the case of permissible* NNs, the message ceases to be disrespectful. For permissible* NNs, the message—that the RMI has been weakened to the point it can permissibly* be infringed—would be true, and thus not a misrepresentation.

We are assuming here that an NN would be permissible* only if the subject’s RMI had in fact been weakened. This assumption mirrors a common thought regarding traditional criminal justice measures, such as incarceration (Hurka 1982; Kershnar 2002; Nozick 1974, pp. 137–138; Quinn 1985; Wellman 2012).^23^ It is commonly thought that a necessary part of the justification for incarceration is that offenders have forfeited or weakened some of their rights. It is this, many would hold, that explains why incarceration can justifiably be imposed on criminal offenders, but not on otherwise similar non-offenders.^24^ We find it plausible that something similar must hold in relation to NNs, if their imposition were ever to be justified. Note that those who think that NNs are always impermissible can agree with the claim that NNs are justified only if the offender’s rights have been forfeited or weakened in virtue of their having committed a crime. This conditional claim does not imply that, if the offender’s rights *have* been weakened, then it is permissible for the state to implement an NN.

In cases of permissible* NNs, then, the message that the offender’s RMI has been weakened to the point that its infringement is permissible* is not a negative misrepresentation. It neither makes a false claim about the weakening of the right, nor a false claim about the permissibility* of the action. Consequently, the message is not disrespectful.

### 4.3 Attempt 2

The second attempt holds that the administration of NNs expresses not merely that the offender’s right has been weakened to the point that it is permissibly* infringed, but rather, that the offender does not have the relevant right at all. Because of this, the second horn of the dilemma fails. If the offender still has the right (albeit in weakened form), yet the message expressed is that he or she does not, then the message is a negative misrepresentation, and the action is disrespectful. This result holds *even if* the right is permissibly* infringed by the use of an NN; the problem in these cases is that the message *overstates* the situation, implying that the right has been lost when it has merely been weakened. The message thus remains disrespectful.

### 4.4 Response

We agree that, were this to be the situation, the dilemma would fail. However, there are reasons to doubt that NNs do express this message. Moreover, even if they do, the expressivist would then run into another difficulty: her argument would show too much. To see why, notice that actions do not typically express messages explicitly; where they express a message, the messages are usually communicated implicitly. In order to determine the meaning of the message the context will be relevant. To make our point clearer, consider a different right, the right to freedom of movement (RFM). Practices in the context of criminal justice commonly restrict freedom of movement. Examples include parole conditions, which sometimes include limitations on where the offender can and cannot go, house arrest, which limits movement even more, and, of course, imprisonment. What messages do these practices send? Given our discussion, two candidates are obvious: (1) setting expressivist considerations aside, the offender’s RFM has been weakened to the point where it is permissible to infringe the RFM, and (2) the offender no longer has the RFM. The first message is analogous to the one we discuss in the dilemma, the second is analogous to the message that the current objector claims is expressed by NNs.

If such practices express the second message, and offenders in fact still have the RFM, then such actions would be disrespectful. Yet this would make the Expressivist Objection too powerful, since it would imply that much of what criminal justice systems do is disrespectful and thereby wrong. In order to avoid this problem, the expressivist could argue that this is not the message expressed by such practices; rather, such practices express only the weaker message that the right has been weakened.^25^

How might one argue for this? One might begin by pointing out that, although such practices restrict freedom of movement, they do not do so *completely*; offenders in these conditions are still given some control over whether they move, and have a certain amount of space to move within. The more that the state restricts the offender’s freedom of movement, the weaker the right, according to the message sent. For instance, house arrest may express the message that the offender’s RFM has been weakened to some degree, and imprisonment might express the message that the RFM has been weakened to an even greater extent.

Another matter one might consider in this connection is how the state would treat the offender in other circumstances. For instance, suppose it were found that keeping prisoners chained to a chair or in a straitjacket would make them more manageable as prisoners. Would the state do this to incarcerated offenders? Although we don’t mean to imply that states always treat offenders in the way that they should, many states have rejected forms of punishment akin to these, and it seems unlikely that they would implement these punishments. This suggests that not only is the state’s actual behavior limited in how it restricts freedom of movement, the state is also limited in how much it would restrict prisoners in other possible circumstances. This gives us further reason for thinking that practices like house arrest and imprisonment, even if they express a message including the claim that the offender’s RFM has been weakened, do not express the claim that this right has been lost.

However, notice that these same sorts of reasons can be applied to cases of NNs. A neurointervention like chemical castration, for instance, is limited in how much it interferes with bodily and mental integrity. It is intended to target a specific subset of mental states, and only a part of how the body functions. Likewise, it is doubtful that the state would behave in ways that would be permissible only if the offender had lost the right. For instance, if it turned out that physical castration was cheaper, or more effective, than chemical castration, it is unlikely that the more enlightened liberal states would employ it. Or, at least, the state need not be so disposed.

The state’s actual treatment of the offender, as well as how the state would treat the offender in different circumstances, help us to see what the meaning of the action is, given that actions do not typically express the content of messages explicitly. If the state’s actual behavior is limited, *and* the state would not further infringe the relevant right in ways that would only be permissible were the offender to not have the right, we have reason to think that the message expressed includes the claim that the relevant right is weakened, but not the claim that it is lost. These points help the expressivist evade the problematic message when it comes to practices that infringe the RFM, yet they also help to avoid a similar message about the RBI and the RMI.

The expressivist may, at this point, attempt to appeal to infringements of the RBI and the RMI in the past. Consider, again, the use of physical castration. In the past, some states nonconsensually imposed physical castration on certain criminal offenders. These past actions, the expressivist might claim, are the ones that set the meaning of NNs, and that meaning includes the claim that the relevant right has been lost. Given the Expansion Thesis, this meaning has spread to current NNs, and will expand to future NNs. Thus, even current, or future, practices involving NNs express the stronger message.

We have two points to make in response. First, one could take a similar line with the RFM, and suggest that current practices of house arrest and imprisonment express the claim that the offender’s RFM has been lost. Just as it is true that, in the past, the state’s practices concerning NNs were quite different from the practices being employed or considered today, so too were their practices concerning restriction of movement. For instance, some states shackled prisoners to balls and chains, or they placed prisoners in stocks and pillories, which severely limited freedom of movement. Thus, if this objection proves that the message expressed by NNs was set by these older, much different, practices, it is unclear why we do not get the same conclusion for practices that infringe the RFM. Again, we think, this message would be too strong.

Second, recall that for an action to be disrespectful, it must express a message that is a negative misrepresentation of the subject, *as appropriately understood by members of the community.* Even if the expressivist offers a response to the previous point, one might reasonably ask why the message that the offender’s relevant right has been lost is an appropriate understanding of the action, in light of the points made above about current practices. Given that the current or future use of NNs would (or could) be limited in scope, and given that the state is not disposed to infringe the relevant right in various other circumstances, it is difficult to see how an appropriate understanding of these actions would include the claim that the right has been lost, rather than merely weakened.

### 4.5 Attempt 3

A third attempt to escape the dilemma maintains that the message expressed by NNs concerns not only the RMI and the RBI, but also a further right: the right to define a boundary around oneself that no-one may transgress. According to Shaw, actions that interfere with *both* the body and the mind express a “*clear and thoroughgoing* disregard for the victim’s right to draw a boundary around an area of her life with which the other party must not interfere” (Shaw 2018a, p. 334). If NNs indeed infringe this right to draw a boundary, one might worry that they also express the message that the offender does not have this further right.

### 4.6 Response

We begin by noting that, even if we have this right to draw a boundary around an area of one’s life with which the other party must not interfere, this right surely has limits. Our income is an area of our lives that we would want to draw a boundary around, yet we do not have a right to draw a boundary around it such that the government cannot take some of it through taxation. Likewise, freedom of movement is an important part of most of our lives, yet even if an offender wants to draw a boundary around it, it may still be permissible for the state to restrict the offender’s movement—at least, those who accept even the most minimal forms of incarceration seem committed to this. Given that this right, if we have it, has limits, and given that these limits can apparently change on the basis of past offenses, we would need more argumentation for the claim that offenders have the right to set a boundary that would rule out NNs. If they do not, then it is not clear why expressing the message that they lack such a right would be disrespectful.

Rather than appealing to the offender’s right to draw this boundary, the expressivist could instead rely on the claim that there is an objectively justified boundary, drawn independently of the individual’s wishes. Perhaps this is what Shaw has in mind when she states that.

[A]n offender, by committing an offence, becomes morally liable to interference by the state. The area of the offender’s life that the state must not invade becomes narrower (how narrow it becomes will depend partly on the gravity of the offense). However, all offenders, even while being punished, retain the status of being separate individuals, worthy of a fundamental level of respect. In order to retain this status … there must be some area of the offender’s life from which the state is excluded. The most basic areas, which the offender has the strongest right to control, are the mind and body - because, as argued above, the mind and body are constitutive of personhood. (Shaw 2018a, pp. 333–334)

On this view, it is the fact that the offender is a person, or of equal status to normal persons, that establishes a boundary that must never be crossed; a boundary circumscribing an inviolable core. Although it is true that, by offending, an agent can shift the edges of this boundary, there is still a limit to how much the boundary can shrink. Actions that infringe both the RMI and the RBI, such as NNs, would cross this boundary. This fits well with the thought that the mind and the body are constitutive of the person, and mental and bodily integrity “are necessary in order for someone to have the status of being an agent at all” (Shaw 2018a, p. 323). Thus, expressing the message that it is permissible* for the state to infringe these rights implies the further message that the offender has no inviolable core.

This line of objection presents a problem for the proponent of the expressivist argument, since, on this picture, the expressivist argument again becomes redundant.

The message that the offender does not have an inviolable core, as well as the message that the RMI and RBI are permissibly* infringed, would be negative misrepresentations, but this is because it is never permissible* for the state to perform actions that infringe both the RMI and the RBI. Further, relying on this picture closes off the possibility of rescuing the argument by appealing to the Expansion Thesis. On this picture, there are no permissible* NNs (at least not on persons), since, again, infringing both the RBI and RMI is never permissible*. Thus, there are no other cases for the meaning to expand to. Neither version of the appeal to a boundary, then, seems to be a promising route for the expressivist.

### 4.7 Attempt 4

Finally, a fourth attempt to escape the dilemma appeals to the way in which disrespect can aggregate. When discussing NNs, it is important to consider individual actions, as well as individual rights that may be infringed. Yet, if we limit our focus to these, we will fail to see a critical moral feature of employing NNs. One might think that “mandatory neurointerventions vividly express disrespect because of the cumulative effect of the actions of which they consist” (Shaw 2018a, p. 334). The disrespectful nature of NNs arises not from some particular action or other, nor from some particular right infringement or other. Rather, the disrespect arises from the cumulative effect of the state’s course of conduct taken as a whole; say, a series of administrations of the drug, or multiple rights infringements.

### 4.8 Response

There are multiple ways to account for this disrespect, yet none of these, we argue, will help the expressivist escape the dilemma. On one way of developing this objection, the disrespect arises from the infringement of multiple rights. A particular action becomes disrespectful in virtue of infringing multiple rights, or perhaps a specified set of rights. This may be what Shaw has in mind when she states that “[t]orture (like mandatory neurointerventions) involves multiple rights violations” (Shaw 2018a, p. 334). Torture, she thinks, is disrespectful, at least in part, because it “involves multiple rights violations” (Shaw 2018a, p. 334) and it represents a “systematic attack on the individual’s rights” (Shaw 2018a, p. 335). On this route, the disrespectful message may be that *both* the RBI and the RMI have been weakened to the point where it is permissible* to infringe both.

Although this is a slightly different message than the one we considered when presenting the dilemma, it does not provide new resources for avoiding it. If it is, in a particular instance, in fact permissible* to infringe both the RMI and the RBI, then the message is not a misrepresentation, and the action is not disrespectful. If, on the other hand, it is impermissible* to infringe both these rights in a particular case, then the message *is* a negative misrepresentation, but the argument becomes redundant; arguing for the claim that it is a misrepresentation requires arguing for the claim that the NN is impermissible*.

One might instead account for the disrespect by suggesting that we should focus on multiple actions and/or omissions. For instance, if some NN requires injections once a month, then, in implementing the NN, the state will perform multiple actions. Even in a case where we have a one-time injection with long-lasting effects, the state’s failure to reverse the intervention could be an omission. One might think that, once enough individual actions and omissions that express *some* disrespect are performed, the disrespect adds up to an amount that makes the action impermissible.

We think this line is not promising. For the objection to work in this way, the individual actions need to be somewhat disrespectful; otherwise, there is no disrespect to add up. But it is not clear that the individual actions *are* disrespectful; we could apply the dilemma to these individual actions, once a particular message is specified.

On a third way of developing this objection, it is not the individual actions and/or omissions that are disrespectful; rather, it is the course of conduct. One might think that “[s]ometimes a number of actions that each infringe another person’s rights can, when considered together, amount to a distinctive type of violation which is more than just the sum of each of the individual infringements” (Shaw 2018a, p. 322). In relation to this point, Shaw presents an analogy with harassment. Consider two sets of actions: (1) send ten unwanted gifts to one individual, and (2) send one unwanted gift to each of ten individuals. Assuming that every individual action produces the same amount of distress on the victim, there seems to be something worse about the first course of conduct, an instance of harassment, than the second course of conduct. In both the case of harassment, and the case of an NN, the course of conduct expresses the message that “the individual who is subjected to such treatment is not entitled to control a certain personal domain” (Shaw 2018a, p. 333).

Alternatively, it may be ambiguous whether the individual actions and/or omissions express this message, yet when considered as a whole, the course of conduct unambiguously expresses this message. Perhaps each individual component of the course of conduct adds to the evidence that this message is being expressed.

These ways of developing the objection do not help the expressivist avoid our arguments above. The content of this message is similar to others we have considered above; namely, messages concerning the right to *some* control over how the state treats the offender, the right to *full* control over how the state treats him, and the right to draw a boundary. What we originally said in response to those messages applies here as well. The shift to claiming that it is the course of conduct that is disrespectful, rather than the individual actions, does not help either; the dilemma applies equally well to courses of conduct as it does to individual actions.

## 5 Concluding Thoughts

We have assessed Elizabeth Shaw’s Expressivist Objection to non-consensual neurointerventions (NNs), according to which such neurointerventions are invariably disrespectful, and invariably wrong, by virtue of the negative message that they express about the recipient of the intervention. We have examined various ways of understanding the message expressed by NNs, dividing such messages into two types. Of the possible capacity messages considered, we argued that none is such that both (i) NNs always send this message, and (ii) sending this message is always disrespectful. We made a similar point regarding a variety of status messages before presenting our dilemma for expressivist arguments that rely on the expressions of a status message.

According to our dilemma, if the status message expressed is true, then it is not disrespectful, and the expressivist argument fails. If, on the other hand, the status message is false, it may be disrespectful. But, arguing for the claim that the message is false requires a non-expressivist argument for the claim that NNs are impermissible*. That is, the proponent of this argument needs to argue that, setting expressivist considerations aside, the balance of moral reasons for and against performing the action already recommends that we should not do it.^26^ Further, and importantly, the expressivist reason against administering the NN is only present in cases where the NN is impermissible*. Consequently, the expressivist argument becomes redundant.

Notice that, given this conclusion, our dilemma will apply also to variants of the Expressivist Objection that are much weaker than Shaw’s own objection, provided that they adopt the same ‘negative misrepresentation’ understanding of “expressed disrespect”, and depend on the claim that a status message is expressed. Indeed, it will apply even to a very weak version of the Objection according to which expressed disrespect *sometimes* makes a difference to the permissibility of NNs. Our responses to attempts 1 and 2 in the previous section give reason to reject the expansion thesis, on which the expressed disrespect expands to NNs that do not violate the relevant rights. If disrespect does not so-expand, then the only cases involving expressed disrespect will be cases in which the NN is already impermissible*. If so, then expressed disrespect will never make a difference to the permissibility of an NN.

On the other hand, our arguments will not apply to variants of the Expressivist Objection that employ different understandings of “expressed disrespect”—such as understandings on which the claim that an action expressed disrespect does not entail the claim that a negative misrepresentation was expressed. However, there are reasons to doubt that such objections would count decisively and universally against NNs. Even if it could be shown that, on some alternative account of disrespect, NNs are invariably disrespectful, the expressivist would face a further challenge. Suppose someone proposes an expressivist argument that makes use of a different account of disrespect. For instance, suppose that an action is disrespectful if it expresses a negative message about a person, *even if* the message is true. Further suppose that this argument succeeds in establishing that all NNs are disrespectful in virtue of expressing negative messages. This would give us some reason to refrain from administering NNs. Yet, one might still wonder whether this reason is sufficiently strong to make it all-things-considered wrong to perform such actions. To answer this latter question, we need to look at other features of the action relevant to its permissibility—features that might affect the strength of the reason not to express the negative message, or the strength of countervailing reasons *to* perform the action that would express the message.

Return to our discussion of local rationality messages (LRM), for example. Assuming that a negative LRM is true—that the agent’s rationality *is* flawed in some way or other—we find it difficult to see how the fact that an action expresses this message could make it all-things-considered wrong, even if it gives us some reason not to perform the action. There are, for instance, many cases where it seems that it is at least permissible to express a negative but true judgment about a person’s rationality. If we think that a friend, who is deliberating about a big decision, is not recognizing the correct reasons, or not giving them the appropriate weight, we may think it permissible to point this out. Even if there is something intrinsic to the communication of such a message that gives us reason not to express it, the reason does not seem to be sufficiently strong to make the expression, or the action that expresses this, impermissible. In fact, it seems that various other unobjectionable measures employed by our criminal justice systems would express this same message. Consider, for instance, talking therapy aimed at rehabilitating the offender. Implementing a talking therapy may well suggest that the subject’s ability to recognize and/or react appropriately to reasons against committing the sort of offense he committed is in some way faulty, and in need of repair. After all, effecting such repair would be the intended purpose of such therapy.

In order to argue that NNs are always impermissible, then, we will need more than arguments for the claims that (i) NNs are disrespectful, and (ii) this gives us *some reason* not to perform the action. We will need an argument for the claim that this reason will always establish impermissibility.

## Data Availability

Not Applicable.

## Declarations

1 We assume here that rehabilitation often has broader goals than merely the prevention of recidivism. For example, it may seek also to facilitate moral reform, to treat mental illness, or to prevent anti-social behavior that is not criminal. For discussion of the nature of rehabilitation, see McNeill (2014); Raynor and Robinson (2005).

2 For discussion of the scientific possibilities, see Chew et al. (2018); for discussion of various objections to such use of neurocorrectives, see Ryberg (2020, Chapters 3–4).

3 Chemical castration can be imposed as part of a criminal sentence in several jurisdictions (Forsberg 2018; Forsberg and Douglas 2016).

4 We have followed Shaw in characterising the Expressivist Objection as a universal objection to NNs. However, our arguments are equally effective against a version of this argument that concludes with the claim that NNs are wrong in virtue of the disrespect they express *except* in extreme cases. Moreover, our core argument, presented at the end of Sect. 3, applies to *any* version of this objection appealing to the disrespect expressed in virtue of negatively misrepresenting the moral status of the subject. We will return to this point in the conclusion.

5 See, for discussion of some such objections Duff (2005).

6 Shaw herself also raises a different expressivist objection to NNs; she suggests that NNs might be problematic by virtue of expressing a grandiose or hubristic attitude on the part of the state (Shaw 2011, pp. 197–198; 2014, p. 13). We neither address this worry, nor regard is as a version or aspect of the Expressivist Objection, since it is doubtful that it can be understood as maintaining that NNs are disrespectful of those whom they target. (The worry here is arguably about a positive misrepresentation of the state, not a negative misrepresentation of the offender.).

7 We will, in our discussion below, sometimes speak of rights infringements and sometimes of rights violations. In contrast to Shaw’s usage, we do not use these terms interchangeably. As we use the term “infringe,” it does not imply moral impermissibility; an action can infringe a right, yet do so permissibly. Shaw refers to what we call rights infringements as rights violations (see, for instance, Shaw (2018a, p. 323)), however we reserve the term “rights violation” for an impermissible rights infringement.

8 This objection is similar to one considered by Ryberg (2020, pp. 129–132).

9 On at least one interpretation, the value one *should* be accorded could be different than one’s actual value. However, as we read Hampton, she intends “the value one should be accorded” to refer to one’s actual value (Hampton 1991, pp. 1674–1677). We interpret Shaw in this fashion as well. She states that “we ought not represent individuals as having less value than they have” (Shaw 2018a, p. 325) and, when explaining the notion of expressed disrespect, she suggests that “[b]y treating the victim as if she were less valuable than she is, the behaviour ‘says’, falsely, that she is less valuable” (Shaw 2018a, p. 323).

10 We do not see how taking the broader interpretation would be objectionable. Any behavior that counts as disrespectful on Shaw’s view will count as disrespectful on ours. Yet, broadening the scope of disrespectful behavior allows us to consider some actions whose meaning may not be about the subject’s value (see Sect. 2).

11 See, for instance, her discussion of the corpse desecrator (Shaw 2018a, p. 326).

12 On some popular views of control, the features of rationality that we focus on are going to be some of the main components of control (see, for instance, Fischer and Ravizza (1998, Chapter 3)). On some other popular views, control, or at least the control required for moral responsibility, is understood in terms of certain abilities, some of which are not constitutive of rationality (e.g., the ability to do otherwise).

13 We borrow this distinction between receptivity and reactivity from Fischer and Ravizza (1998, pp. 41–42), who offer these notions as a part of an account of the control required for moral responsibility. Their view is stated in terms of *sufficient* reasons to perform or omit some action. On our understanding of “action,” practical decisions—decisions about what to do—are (mental) actions.

14 Unlike the previous LRM we considered, this message might be one that *consensual* neurocorrectives do not express. It would seem that part of what justifies the claim that this message is expressed is that, by implementing the NN without the offender’s consent, the state is making this decision, and change, for the offender.

15 There are other potential status messages available. One might think that NNs express the message that the subject is not a (separate) person (for related points, see Shaw (2011, pp. 196–199; 2014, p. 12; 2018a, pp. 329, 333)), or that the subject is not a member of the community (for related points, see Shaw (2011, pp. 188, 198; 2014, pp. 12–13; 2018a, p. 336)). Both of these, we think, can easily be understood in terms of rights. The claim that the subject is not a (separate) person can be understood as the claim that the subject does not have the same rights (either in terms of content or strength) as other, typical persons; and the claim that the subject is not an equal member of the community can be understood as the claim that the subject does not have the same rights as typical members of the moral community.

16 A similar discussion can be found in Shaw (2011, pp. 198–199; 2014, p. 13).

17 For a similar response to this point, see Holmen (2018, p. 222).

18 For a similar point concerning the message that subjects are “sub-human”, see Ryberg (2020, p. 130).

19 One might think that the message sent is not that the RBI and RMI are *weakened* by the commission of a crime, but that an exception clause in these rights is activated such that the NN is now permissible, setting aside expressivist considerations. (It may be, for example, that the RBI protects against a broad range of bodily interferences *except* in individuals who have committed serious crimes and can be prevented from committing further such crimes through bodily interference, in whom it protects only against a narrower range of bodily interferences.) We use ‘weakened’ broadly such that, if, in a particular context, the right provides less protection than it normally would, the right has been weakened. On this reading, activating an exception clause of the sort described above is just one way of weakening a right; one way to make it such that the right does not protect the holder as much as it would protect normal holders of this right in normal circumstances. Readers unhappy with this use of “weakened,” one can instead understand the message as “In this context, the RBI is not such that it makes the action impermissible*,” where the context can include past actions by the rights-holder.

20 One might worry there is an illicit slip here, moving from the claim that the expressivist must argue for the claim that NNs constitute an impermissible rights infringement, *setting aside expressivist considerations* (the claim that they are impermissible*), to the more general claim that they must argue that NNs constitute an impermissible rights infringement. We do not take this to be a problematic feature of our dilemma. As the expressivist argument is intended to work, the fact that the message is disrespectful gives us a moral reason not to perform an action that expresses it. If, expressivist considerations aside, the balance of moral reasons *already* implies that one should not perform the action, adding further moral reasons against performing the action will not change what the overall balance of moral reasons implies regarding permissibility.

21 Earlier, we mentioned the possibility that the content of the message expressed will be partly an empirical matter. If the dilemma generalizes to other status messages, the empirical question will be moot.

22 In detailing this part of her view, Shaw claims that the meaning expands to other actions of the same type, and references Dan-Cohen’s view in this connection. We follow Dan-Cohen and limit the expansion to other actions of the same type, *in the same social practices* (Dan-Cohen 2002, pp. 162–163). This further qualification is required to avoid objections concerning actions of similar type in other contexts (e.g., although physical castration may be disrespectful in the context of punishment, it would not be disrespectful in at least some medical contexts).

23 Some of these theorists offer views on which punishment is justified by the fact that the offender has forfeited, or lost, some of their rights, rather than merely weakened them. However, the rights often discussed are rather narrow rights–such as the right not to be punished (Wellman 2012) or the right to liberty-for-ten-years (Kershnar 2002). One might think that these narrow rights are parts of, or derive from, broader or more fundamental rights, and that those broader or more fundamental rights are not lost, but are merely weakened. So, for instance, an offender might retain an, albeit weakened, RMI while having lost the right to integrity of a particular subset of mental states.

24 We return to this later, but Shaw herself seems to agree with the general point, claiming that by offending, and offender makes oneself morally liable to interference by the state (Shaw 2018a, p. 333).

25 One may wish to avoid this problem by accepting the claim that these practices restricting freedom of movement are disrespectful, yet denying that this claim gets us the conclusion that the practices are all-things-considered impermissible. We return to consider the view that disrespectful actions are sometimes permissible in the conclusion. However, notice that, on this view, the Expressivist Objection fails, given that one will no longer be able to argue from the claim that all NNs are disrespectful to the claim that all NNs are all-things-considered impermissible.

26 Someone might think that this claim about the balance of reasons is different than a claim about impermissibility (or impermissibility*). This would be because, in order for an action to be impermissible, the balance of reasons needs to be *significantly* against the action. We could state our point using this formulation as well.
